# Fertility Preservation in the Era of Immuno-Oncology: Lights and Shadows

**DOI:** 10.3390/jpm14040431

**Published:** 2024-04-19

**Authors:** Erica Silvestris, Stella D’Oronzo, Easter Anna Petracca, Claudia D’Addario, Gennaro Cormio, Vera Loizzi, Stefano Canosa, Giacomo Corrado

**Affiliations:** 1 Gynecologic Oncology Unit, IRCCS Istituto Tumori “Giovanni Paolo II” Bari, 70124 Bari, Italy; easteranna97@hotmail.it (E.A.P.); gennaro.cormio@uniba.it (G.C.); vera.loizzi@uniba.it (V.L.); 2 Department of Interdisciplinary Medicine (DIM), University of Bari “Aldo Moro”, 70121 Bari, Italy; claudia.daddario@uniba.it; 3 Division of Medical Oncology, A.O.U. Consorziale Policlinico di Bari, 70124 Bari, Italy; 4 Department of Translational Biomedicine and Neuroscience (DiBraiN), University of Bari “Aldo Moro”, 70124 Bari, Italy; 5 IVIRMA, Global Research Alliance, LIVET, 10126 Turin, Italy; stefano.canosa@unito.it; 6 Gynecologic Oncology Unit, Department of Woman, Child Health and Public Health, Fondazione Policlinico Universitario A. Gemelli, IRCCS, 00136 Roma, Italy; giacomo.corrado@policlinicogemelli.it

**Keywords:** assisted reproduction, fertility preservation, infertility, reproductive medicine

## Abstract

In recent years, immuno-oncology has revolutionized the cancer treatment field by harnessing the immune system’s power to counteract cancer cells. While this innovative approach holds great promise for improving cancer outcomes, it also raises important considerations related to fertility and reproductive toxicity. In fact, most young females receiving gonadotoxic anti-cancer treatments undergo iatrogenic ovarian exhaustion, resulting in a permanent illness that precludes the vocation of motherhood as a natural female sexual identity. Although commonly used, oocyte cryopreservation for future in vitro fertilization and even ovarian cortex transplantation are considered unsafe procedures in cancer patients due to their oncogenic risks; whereas, ovarian stem cells might support neo-oogenesis, providing a novel stemness model of regenerative medicine for future fertility preservation programs in oncology. Recent scientific evidence has postulated that immune checkpoint inhibitors (ICIs) might in some way reduce fertility by inducing either primary or secondary hypogonadism, whose incidence and mechanisms are not yet known. Therefore, considering the lack of data, it is currently not possible to define the most suitable FP procedure for young patients who are candidates for ICIs. In this report, we will investigate the few available data concerning the molecular regulation of ICI therapy and their resulting gonadal toxicity, to hypothesize the most suitable fertility preservation strategy for patients receiving these drugs.

## 1. Introduction

Infertility is a relevant issue in our society today, with a demographic, social, and medical impact. According to the World Health Organization (WHO), a significant increase in the number of infertile couples has been reported, reaching 48 million, with 186 million males and females affected by infertility [1]. Infertility is the inability to conceive after sexual intercourse during a period of one year with approximately 2–4 attempts per week, without any contraceptive method [1]. The rise in infertility can be attributed to sociocultural changes that have led to the postponement of decisions regarding parenthood, as well as cases of skepticism in recognizing the existence of a reproductive problem. Furthermore, in line with increasing age, detrimental changes in gonadal function occur, resulting in a loss of quality and quantity of ovarian reserves in women, as well as decreasing testosterone levels in men [2].

Most female patients undergoing anti-cancer care develop cancer treatment-related infertility (CTRI) as an effect of oocyte desertification in their ovaries after receiving gonadotoxic drugs, as well as those undergoing radiation treatments, which result in permanent illness that precludes projects of normal female reproductive life [3]. In fact, even after cancer treatment and recovering from cancer, young patients, in particular women of a procreative age, experience severe psycho-social distress and depression due to the woman’s negation of their primary sexual identity as a mother [4]. 

Although gonadotropin-releasing hormone analogues (GnRHa) partially reduce the level of ovarian damage [5], the clinical standard of fertility preservation (FP) in females with cancer is based on transvaginal oocyte retrieval, cryopreservation for future in vitro fertilization (IVF), and subsequent intrauterine embryo transfer. However, in order to retrieve mature eggs this procedure employs estrogen boosters to promote multiple follicular growth and needs a time-dependent maturation of oocytes requiring up to two weeks. Thus, for the intrinsic oncogenic effect, this is a risky procedure, particularly in adolescent patients with hormone-sensitive cancers [6] and is inconvenient in cases of urgent neoadjuvant treatments required for aggressive tumors [7], or in those of a prepubertal age [8]. Other strategies include autologous transplantation of cryopreserved cortical tissue strips after completion of cancer treatment [9], as well as the collection of immature oocytes at their germinal vesicle stage for subsequent in vitro maturation (IVM) and IVF [10]. Nevertheless, besides the additional oncogenic risk induced by hormone stimulation, most of these techniques are inappropriate for cancer patients in relation to their empirical retrieval and operator-dependent selection of viable eggs. Also, the cortical strips’ reimplantation is fully operator-dependent and even in healthy women results are variable, with a modest success rate in pregnancy; devoted studies show that their highest value of pregnancy rates recorded is lower than 40% [11]. Moreover, this procedure is unsafe for potential replacement in ovaries with blood-born malignant cells, as leukemia or cancer cells originally reside in the frozen cortex [12]. Next to the above-mentioned FP procedures, another interesting approach is the isolation of ovarian stem cells (OSCs) from the ovarian cortex.

In recent years, the human oocyte has gained significant attention from researchers as they have tried to better understand the most crucial cell in female mammalian reproduction and they wish to, ultimately, obtain fertilizable oocytes through in vitro methods. It has been demonstrated that oocytes, also known as germ cells, have an extragonadal origin, established during embryogenesis from somatic cells before organ systems’ development. Germline precursors are represented by primordial germ cells (PGCs). Depending on the gonadal environment, PGCs have the potential to develop into either oocytes or spermatogonia [13]. Once PGCs reach the female gonad, they are referred to as oogonia, which undergo a limited period of mitotic proliferation before progressing into meiosis and forming oocytes [14]. At the diplotene stage of prophase I, oocytes become enclosed within somatic cells known as granulosa cells, resulting in the formation of primordial follicles. The formation of primordial follicles is responsible for establishing the follicles pool and several factors, including the balance between oocyte survival and death during this phase, determine its size. This process, in turn, will determine the ovarian reserve and fertility [13].

Recently, the central dogma that the number of oocytes is fixed during a woman’s lifetime [15] has been questioned, opening up the possibility of exploring collect and expand in vitro OSCs before therapies, in order to obtain large oocyte-like cells (OLCs) that can be frozen to constitute the individual oocyte reserve for each young cancer patient [16].

The preservation of reproductive function is crucial for the long-term well-being of cancer survivors, regardless of gender; hence, it is relevant to highlight that male fertility can also be affected as well as their female counterparts’. According to the current guidelines from the American Society of Clinical Oncology (ASCO) [17], it is recommended to discuss the FP procedures available for young male oncological patients, including sperm cryopreservation. With regards to this well-established procedure, several studies reported a successful sperm collection already in those as young as 12 years [18]. Assisted reproductive treatments, such as IVF and intracytoplasmic sperm injection (ICSI), which employ cryopreserved semen before gonadotoxic treatment, can lead to good pregnancy outcomes. The impact of cancer treatment on prepubertal patients primarily affects proliferating Sertoli cells, thus impairing the seminiferous tubules’ (STs) growth [19].

STs are the functional unit of the testis and occupy two-thirds of the organ’s volume. They consist of a basement membrane, Sertoli cells (SCs), and germ cells at various stages of development. SCs represent around 17–20% of the STs’ epithelium in adult men and play a crucial role in testis’ formation and sperm production. Thus, their number determines the testis’ size and the amount of daily sperm production [20]. As mentioned above, during the first six weeks of gestation, PGCs can become either male or female. The presence of the Y-chromosome or, specifically, the SRY gene, determines the development of reproductive organs into testes [13]. Within the STs, germ cells are those intended to ultimately become spermatozoa. Firstly, between 3 and 5 weeks of development, they become gonocytes under the influence of SC factors. Then, they continue to differentiate into spermatogonia (SPG) until up to 6 months, when they become quiescent until the age of 5–7 years. After that, once puberty occurs SPG increase in number with the dual role of undergoing meiosis to produce spermatozoa, and mitosis occurs to ensure continuous sperm production throughout a man’s life. These processes take place in the basal compartment of STs and involve close interaction with SCs [20]. 

Consequently, when SPG are lost, spermatogenesis cannot be restored, and fertility impairment occurs. Since the assessment of spermatogenesis can only be evaluated through semen analysis, an alternative option for prepubertal males is testis tissue cryopreservation (TTC). As well as ovarian cortex cryopreservation, testicular tissue can be obtained by biopsy and preserved employing slow-freeze or ultra-rapid techniques. After thawing, TTC could be grafted to testis for autologous transplantation of SSCs or adopted for in vitro spermatogenesis [17]. 

Today, only a few studies concerning the impact of immunotherapy on male fertility are available, with just one trial in vivo showing that treatment does not seem to affect fertility, but spermatogenesis [21]. However, therapies’ effects on male patients still needs to be elucidated; hence, in this report we focus on the impact of immune checkpoint inhibitors (ICIs) on females’ fertility and the new strategies for future FP.

In the last decade, the advent of immunotherapy in oncology has led to the routine use of some anti-cancer treatments, specifically immune checkpoint inhibitors (ICIs), such as the anti-PD-1/anti-PDL-1/anti-CTLA-4 antibodies, which significantly improved the prognosis and recovery rates in patients suffering from melanoma, as well as lung and kidney cancers; although, among the others, currently there are still no data concerning their possible role in compromising fertility [22].

Recent scientific evidence has postulated that ICIs might in some way reduce fertility according to two principal mechanisms: a primary hypogonadism due to an immune-mediated damage to the gonads, whose incidence is also not currently quantifiable [23] and a secondary hypogonadism, defined as a functional impairment of the pituitary–gonadal axis according to a mechanism not yet known [24]. Therefore, considering the lack of data, unfortunately it is currently not possible to define indications for young patients who are planning to start treatment with anti-PD1/anti-PDL1/anti-CTLA4 drugs (such as nivolumab, pembrolizumab, atezolizumab, ipilimumab, etc.) and who wish to preserve their fertility. 

Therefore, in this report, our aim is to investigate the few available data concerning the molecular regulation of ICI therapy and their resulting gonadal toxicity, in order to detect the most suitable FP strategy before treatment with these drugs in relation to the patient’s medical history, genetic profile, and reproductive goals.

## 2. Immunotherapy: From Concept to Clinical Application

In 2011, Hanahan and Weinberg listed the capability to evade immune response among the hallmarks of cancer [25], paving the way to a novel therapeutic approach to malignancies, namely immunotherapy, in which the patient’s immune system is induced to exploit a long-lasting anti-tumor response [26].

Already in the 20th century, some biologists had postulated that a functionally competent immune system is constantly surveilling cells throughout the body, potentially recognizing and eliminating incipient cancer cells to arrest the development of nascent tumors [27].

This theory was later supported by the evidence that murine models with defective T/NK cells were more susceptible to cancer [28], and by an increased risk of certain malignancies observed in immunocompromised patients [29].

In addition, tumor microenvironments were shown to be immunosuppressive due to the considerable presence of T regulatory cells (Treg), myeloid-derived suppressor cells (MDSCs), and M2 macrophages that cooperate and help cancer cells to develop multiple strategies for immune evasion [30]. Based on these observations, in the last decade, boosting the immune system has become the major thread for anti-cancer drug development. 

Two categories of immunotherapies have been introduced in clinical practice, defined as “passive” and “active”, respectively. The former category (which includes cytokines, adoptive cell, and antibody-based therapies) consists of molecules that are lowly expressed and not functioning in the patient, and whose expression cannot be induced; the latter category aims at the direct activation of tumor-specific immune responses and is represented by cancer vaccines, oncolytic viruses, and ICIs [26].

ICIs are monoclonal antibodies that work against the “immune checkpoints”, namely those molecules of co-inhibitory signaling pathways that physiologically maintain immune tolerance and are often used by tumor cells to evade immune surveillance; hence, their employment enhances the immune-mediated elimination of malignant cells [31]. ICIs’ main targets are the cytotoxic T lymphocyte-associated molecule-4 (CTLA-4), programmed cell death receptor-1 (PD-1) and its ligand, and programmed cell death ligand-1 (PD-L1). CTLA-4 is a co-inhibitory molecule expressed on T cells that negatively regulates their activation at the initial priming phase. Based on the successful results of a randomized clinical trial, the anti-CTLA-4 monoclonal antibody ipilimumab became the first ICI approved for cancer treatment and was used in the setting of advanced melanoma [32,33]. Later on, ipilimumab was approved for the treatment of other malignancies, including renal cell carcinoma (RCC), non-small cell lung cancer (NSCLC) [34], malignant pleural mesothelioma, etc. In a similar fashion, another CTLA-4 targeting ICI, namely tremelimumab, was approved by the Food and Drug Administration in combination regimens in the setting of HCC [35] and NSCLC [36]. Further anti-CTLA-4 monoclonal antibodies are still under development, aimed at increasing the efficacy of such agents when administered as monotherapies, while reducing the risk of side effects [37].

**Figure 1:**
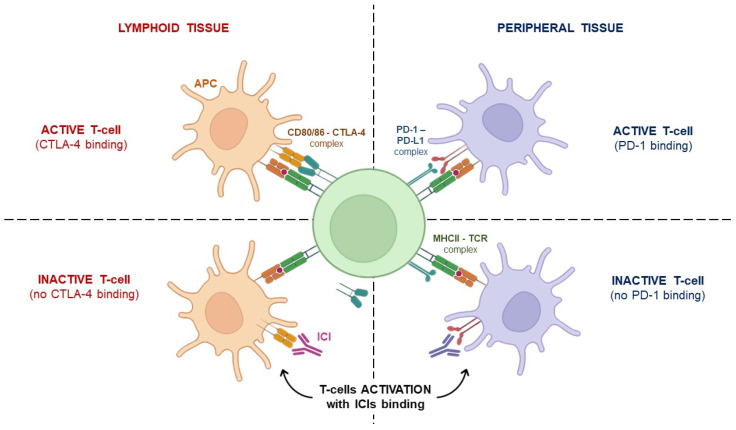
T-cell activation following ICI treatment. T-cell activation is the additional effect of TCR interaction with MHCII on the APC surface, and CD28 surface family proteins (CTLA-4 and PD-1) binding to B7 family ligands, such as CD80/86 and PD-L1. If one of these matches is missing, T cells cannot be activated. ICIs, such as ipilimumab or pembrolizumab, can restore the signaling by mimicking the co-stimulatory signals. TCR: T-cell receptor; MHC-II: Major Histocompatibility Complex Type II; APC: antigen presenting cell; ICIs: immune checkpoint inhibitors.

On the other hand, PD-1 was discovered to be expressed on the surface of all T cells during activation, which is controlled by PD-L1 or PD-L2 binding. If PD-L2 can be found only on monocytes and dendritic cell surfaces, PD-L1 can be expressed even by resting T cells, B cells, and parenchymal cells, and its presence is associated with a poor prognosis in patients next to TILs’ (Tumor-Infiltrating Lymphocytes) evidence [26]. The PD-1/PDL-1 axis regulates the functions of T cell effectors during several physiological responses, including acute and chronic infections, immune homeostasis, autoimmunity, and cancer [38]. In contrast with CTLA-4, which exerts its regulatory effect mainly within lymphoid tissues, PD-1 exerts its function within peripheral tissues (Figure 1) [39]. Several inhibitors of the PD-1/PDL-1 axis have been successfully developed so far, entering the clinical practice in both combination and monotherapy regimens. Among these, it is worth mentioning that the anti-PD-1 nivolumab, pembrolizumab, and cemiplimab, as well as the anti-PD-L1 agents, namely atezolizumab, avelumab, and durvalumab, are currently available for the treatment of about 15 different malignancies thanks to the durable clinical responses observed even in the metastatic setting [40].

Despite the impressive results obtained in cancer patients since the advent of ICIs, over half of them experienced the so-called immune-related adverse events (irAEs), which can have a negative impact on quality of life, potentially cause treatment interruption and/or cessation, and can even be fatal [41]. Such toxicities often mimic autoimmune disorders and can occur in several organs and systems, with the most common observations being in the gastrointestinal tract (e.g., colitis and diarrhea); [42], endocrine system (more frequently thyroiditis and hypophysitis) [43], skin (rash and pruritus) [44], rheumatologic systems (arthritis, myositis, and lupus-like syndromes) [45], as well as in the liver (increased aspartate and alanine transaminases) and lungs (pneumonitis) [29]. In addition, other non-organ-specific events, such as fatigue, have been reported [46], and fatal irAEs have been observed in 0.4–1.2% of patients, with myocarditis being the most common one [47].

A correlation between the onset of irAEs and response to ICI treatment has been hypothesized, but with conflicting results coming from clinical studies [48].

Although long-term irreversible and chronic complications can occur [46,47,48], irAEs are generally reversible and manageable with corticosteroids and immunosuppressive drugs. Their severity is extremely variable and graded according to the common terminology criteria for adverse events [46]. Their management is guided by the recommendations released by the ASCO [46] and the European Society of Medical Oncology (ESMO) [44]. 

Unfortunately, no biomarker exists to predict which patients are more likely to develop irAEs, but it appears that pre-existing conditions (e.g., dysregulation of the gut microbiome, autoimmune diseases, host genetic factors, etc.) may influence such risks [48].

The focus of this review is the impact of ICIs on female fertility which will be discussed in detail in the following session. 

## 3. ICIs’ Impact on Female Fertility

In recent years, the field of immuno-oncology has revolutionized cancer treatment by harnessing the power of the immune system to fight cancer cells. While this innovative approach holds great promise for improving cancer outcomes, it also raises important considerations related to fertility and reproductive toxicity. As researchers delve deeper into the complexities of immuno-oncology, understanding the impact on fertility becomes crucial for individuals facing cancer treatment and the medical professionals guiding them through this journey [49]. Indeed, like other anti-cancer treatments such as chemotherapy and radiation therapy, immunotherapy can potentially have adverse effects on fertility. These effects may be due to direct damage to reproductive organs, hormonal imbalances, or other mechanisms.

**Figure 2:**
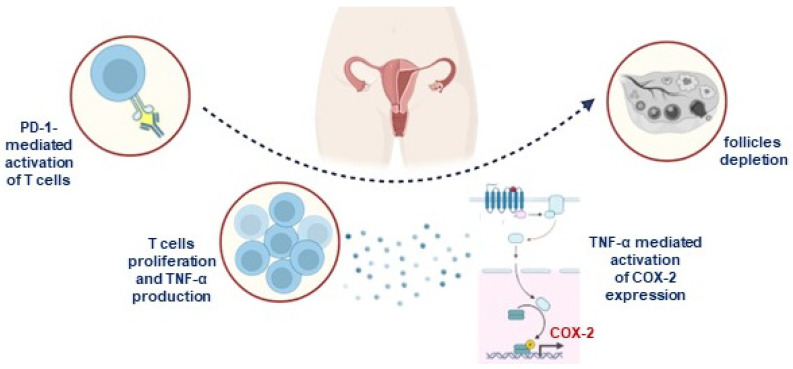
CD3+ T-cell activation and infiltration correlates with TNF-α and COX-2 induction. Mice injected with pembrolizumab showed higher CD3+ T-cell infiltration, particularly near the ovarian epithelium, where primordial follicles are typically located. Together with T-cell density, higher levels of TNF-α were found. Cytokine signaling seems to increase COX-2 transcription and, together with TNF-α, create a lethal pro-inflammatory microenvironment for follicle maturation. COX-2: cyclooxigenase-2.

Immune checkpoint inhibitors, a cornerstone of immuno-oncology, work by releasing the brakes on the immune system, enabling it to recognize and attack cancer cells. While this mechanism is critical for cancer treatment, it can inadvertently affect reproductive tissues. The reproductive function of patients treated with ICIs can be directly influenced by dysfunctions at the gonadal level or, indirectly, by dysfunctions at the hypothalamic and pituitary levels [50]. The first mechanism of hypofertility could reside in primary hypogonadism, due to immune-mediated damage to the gonads. Although this effect appears possible, its real impact is not currently known. However, several preclinical studies based on animal experiments have proposed that ICIs have a direct effect on female gonadal function and have analyzed their mechanism of action. Preclinical studies conducted on monkeys treated with ipilimumab have shown that the antibody binds to the ovarian connective tissue without causing changes in the histopathological morphology of the oocyte [51]. Toxicity studies at doses six times the recommended dose of atezolizumab in female monkeys resulted in irregular menstrual cycles with a lack of corpora lutea [52]. For pembrolizumab, the numbers of primordial follicles were significantly higher after injection of the drug, as observed in a murine anti-mouse PD-1 antibody injected into prepubertal immunocompetent female mice. On the other hand, no change in the number of follicles was observed in immunodeficient nude mice. Furthermore, the researchers found increased TNFα following the up-regulation of cyclooxygenase-2 in the ovaries following administration of anti-mouse PD-1 antibodies, as well as infiltration of CD3+ T cells within some follicles and between ovarian stromal cells (Figure 2). The authors of the study concluded that the blockade of the PD-1 immune checkpoint affects the ovarian reserve by a mechanism that involves the infiltration of CD3+ T cells. This is the first study to demonstrate a link between ICIs and inflammation-mediated follicular depletion in preclinical prepubertal mouse models [53]. In a recent pre-clinical study by Winship et al. [54], researchers used both tumor-bearing and tumor-free adult female mice to evaluate the effects of PD-L1 and CTLA-4 on the ovaries. This study demonstrated that the depletion of ovarian follicles by ICIs is mediated by immune cells, and found that ICIs increased the infiltration of immune cells and the expression of TNFα in the ovaries, resulting in a reduction of the follicular reserve and the impairment of the ability of oocytes to mature and ovulate. However, further studies in women are needed to validate these findings and investigate whether this phenomenon is also present in humans. To date, we can argue that ICIs have the potential to cause immediate damage to fertility in female cancer survivors, suggesting the need to perform further studies in this field and to preserve fertility in women with cancer undergoing such treatments.

The second mechanism of hypofertility may reside in the indirect effects of hypophysitis on reproductive functions. Immune-related endocrinopathy caused by ICIs is one of the most frequent irAEs, with examples including hypophysitis, primary thyroid disease, primary adrenal insufficiency, and diabetes mellitus. Unlike other irAEs, these kinds of disorders are usually irreversible and require lifelong hormone replacement therapy [55]. A recent analysis of VigiBase, a global database of the WHO, found that endocrine diseases caused by ICIs are reported more frequently, more so in combination therapy [56]. The main clinical consequence of ICI-related hypophysitis is deficiency of one or more pituitary hormones such as the thyroid stimulating hormone (TSH), the adrenocorticotropic hormone (ACTH), the follicle stimulating hormone (FSH), and the luteinizing hormone (LH) [57]. Since the pituitary gland plays a key role in the regulation of the ovaries and testes, its dysregulation can lead to serious damage such as premature menopause and low testosterone levels with subsequent erectile dysfunction and decreased sperm production. Therefore, secondary hypogonadism and infertility may occur in patients with ICI-induced hypophysitis due to disruption of the hypothalamic pituitary–gonadal (HPG) axis [58]. Ipilimumab is believed to cause hypophysitis through type II and type IV hypersensitivity reactions [59]. 

Studies involving melanoma patients found that 11% of those who received a 3 mg/kg dose of Ipilimumab experienced anterior hypophysitis. This percentage increased to 25% when the dose reached 10 mg/kg. Albarel and colleagues found 86.6% of patients suffered from thyrotrophic deficiency, while 85.7% experienced gonadotrophic deficiency [60]. The former was defined as a low T4 plasma level (<12 pmol/L) with a low or borderline TSH level, while the latter consisted of low plasma sex steroids with inappropriate gonadotrophin levels, amenorrhea in non-menopausal women, or a lack of increase in such hormones in menopausal women. Other studies confirmed central hypothyroidism and low LH and FSH levels, which were below 2.2 IU/L and 6.5 mIU/mL, respectively [61]. However, there are still conflicting data regarding thyroidal and gonadal function recovery.

There is little pathophysiological evidence for anti-PD-1/PD-L1 antibody-induced hypophysitis. Recently, the study by Kanie et al. [62] hypothesized that ectopic expression of ACTH in tumors may lead to anti-PD-1/PD-L1 antibody-induced hypophysitis. 

However, responses to ICIs can vary widely among individuals, and this could extend to their impact on fertility. Factors such as the type of cancer, the specific ICI used, and the patient’s overall health may all play a role.

Another critical issue to consider is the potential teratogenicity of ICIs. The CTLA-4/CD80/CD86 and PD-1/PD-L1 pathways are known to be essential for inducing maternal tolerance and preventing semi-allogeneic fetus rejection [63]. Preclinical studies have shown that ICIs increase the risk of intrauterine death. For example, compared to spontaneous abortion, which occurs in approximately 18% of pregnant mice, anti-PD-L1 antibodies significantly increased the rate of spontaneous abortion to 86% [64]. Therefore, based on recommendations like those encouraged following chemotherapy, it is suggested to avoid conception from 6 to 12 months after completion of ICI treatment [65]. Although ICI is not currently recommended for pregnant women and women of childbearing age, several cases of ICI exposure during pregnancy have been published with favorable fetal outcomes without developmental abnormalities [66]. A recent analysis of VigiBase found no evidence of disproportionate reporting in miscarriages, fetal growth restriction, or prematurity [63]. 

Lastly, physicians need to weigh the risk of ICI-related hypogonadism or infertility versus reducing the risk of disease recurrence before using ICIs, and discuss this with the patient beforehand, as this may affect the patient’s acceptance of this therapy.

## 4. Fertility Preservation Procedures for Immunotherapy-Treated Patients

Since still there is little known about immune-related gonadotoxicity, fertility preservation options should be discussed with health care providers as soon as possible before treatment initiation. Indeed, oncofertility counselling is included in the most relevant guidelines for cancer patients’ management, such as those reviewed by the ASCO [67], and should not be underestimated since it seeks to reduce patients’ stress during their entire therapeutic journey, while improving their quality of life. The ASCO has suggested that the best option to prevent fertility impairment related to cancer therapy is embryo/oocytes cryoconservation, or, if necessary, ovarian tissue cryoconservation (OTC) [67]. 

Oocyte cryopreservation consists in controlled ovarian hyperstimulation (COH), mature oocyte retrieval, and cryostorage. Embryo cryopreservation is made up of the same steps, followed by regular IVF or ICSI of the partner/donor’s sperm, and collection at the 8-cell (morula), or blastocyst stage. The main difference between these two is that the former does not require a sperm’s source, which is instead necessary in the latter [68]. To control and permit contemporary maturation of a high number of oocytes during COH, patients receive exogenous recombinant FSH, sometimes replaced with urinary FSH or administered in combination with GnRH analogues (GnRHa), luteinizing hormone LH, or aromatase inhibitors such as letrozole [69,70].

Since ovarian stimulation takes about 2–5 weeks, cryopreservation is not feasible for women requiring urgent anti-cancer therapy who cannot delay treatment for such a long time. Furthermore, these options cannot be used in prepubescent females who have no mature oocytes available [68]. In those cases, ovarian tissue cryopreservation or GnRHa may represent an alternative option.

OTC consists of removing ovarian tissue and collecting cortical fragments, which are then cryopreserved and orthotopically transplanted after therapy to restore ovarian endocrine function. It has been shown that after 4–9 months, OTC is able to restore ovarian function in over 90% of patients [71]. Currently, it represents the only possibility to preserve fertility in young female patients and, given that it does not involve hormonal stimulation and time for ovarian preparation, some countries already consider it a non-experimental procedure [67]. 

If the above-mentioned procedures are not feasible, as well as in breast cancers’ therapeutic setting, GnRHa may represent another chance for fertility preservation [72]. 

GnRHa consist of gonadotropin-releasing hormone agonists and antagonists, which both preserve gonadal function downregulating gonadotrophins production, and thus ovarian cellular turnover. 

GnRH agonists, such as Buserelin and Triptorelin, are competitors for gonadotropin-releasing hormone receptors (GnRHRs) on gonadotrophs. Their binding to GnRHR leads to its internalization and desensitization to GnRH (homologous desensitization). The first feedback is an increase in gonadotrophins (LH and FSH) production, the so called “flare-up effect” [73], which might be responsible for an adverse iatrogenic complication, known as Ovarian Hyperstimulation Syndrome (OHSS) [74]. Despite this, the more the receptors are internalized, the less the HPG axis keeps going on and LH and FSH release decreases, leading to a prepubertal-like environment, with no ovarian activity.

GnRH antagonists, such as Ganirelix and Cetrorelix, bind GnRHR but do not activate it, immediately blocking its signaling, as well as gonadotrophins production [73]. 

GnRHa may have a protective effect on ovarian function by a few potential mechanisms, such as reduction in blood flow (so a lower amount of chemotherapeutics reach the gonads), or up-regulation of anti-apoptotic programs directly on germ line or surrounding cells [72]. ASCO [67], ESHRE (European Society of Human Reproduction and Embryology) [75], and ASRM (American Society for Reproductive Medicine) [76] agree in suggesting antagonists’ employment with respect to the first group of agonists, since, contrastingly from the second ones, the latter shorten ovarian hyperstimulation periods and prevent the risk of OHSS, which can even support tumor sustainment, especially in estrogen receptor-positive breast cancer. Because of that, according to the National Comprehensive Cancer Network, GnRH agonists’ benefits and efficacy need further investigation and they cannot be considered a form of fertility preservation [77].

The adoption of ovarian stem cells (OSCs) in female cancer patients allows us to overcome these troubles while offering a safe and novel procedure to prevent CTRI by regenerating oogenesis.

At first, OSCs were identified in mice ovaries [78], where they represented only 0.0014% of all cells. When OSCs were also found in human ovaries, the dogma that the number of oocytes was fixed during a woman’s lifetime [79] was definitively questioned, paving the way to the hypothesis of novel oocytes’ production after birth. As a matter of fact, it has been demonstrated that, once differentiated in vitro to mature oocytes and reimplanted in sterilized female mice, OSCs restore the ovulatory function [80], thus allowing fertilization and the production of offspring. Recent studies requiring human ovarian cortex explanation showed that OSCs can be purified from cortex fragments by immunosorting with reagents against Ddx4 (DEAD box polypeptide 4), a transmembrane germline marker of oogonial lineage [81], in combination with other stemness markers (Fragilis, Stella, OCT4, and SSEA4) [82]. Under appropriate cell culture conditions, human OSCs differentiate to large oocyte-like cells (OLCs) expressing GDF9 and SYCP3 as genes of mature oocytes and completing their meiotic progression to haploid cells, as revealed by the typical DNA content and single signals on chromosomes X and 5 captured by FISH (Fluorescence In Situ Hybridization) [16]. This property is also shared by oocytes during their IVM, in which they resume a metaphase II (MII) shape, typical of meiosis [83].

The above-mentioned techniques are summarized with their pros and cons in Table 1. 

The last-mentioned evidence suggests that implanting oocytes derived from OSCs into the ovaries of young women with CTRI could be a promising approach to restore follicle regeneration, ovulation, and fertility. However, there are concerns about the potential interference of obtaining new oocytes in vitro with the complex genomic imprinting and epigenetic mechanisms necessary for the development of fully competent oocytes. Disruptions or modification of oocyte transcriptomes as the set of mRNAs expressed at a defined stage could have negative effects on their growth, development, and the resulting embryos [84]. The oocyte’s mRNAs pool is correlated with the ability to develop until the blastocyst stage, and microarray platforms can be used to estimate oocyte quality upon the expression profiles of at least 160 different genes. Among them, 29 candidate genes could be responsible for the difference between good and poor-quality oocytes, such as prostaglandin-endoperoxide synthase 2 (PTGS2) and gremlin 1 (GREM1). Such genes’ expression could potentially be used to predict oocyte competence and select higher quality embryos for transfer [85].

The genomic and transcriptomic study of embryos and/or oocytes’ content is not the only way to predict FP success. Artificial intelligence (AI) has emerged as a potential tool to address the various challenges associated with the evaluation of clinical and embryological decision points during infertility treatment. AI involves machine learning (ML), which employs statistical techniques to enhance machines’ performances thanks to prior experiences, and deep learning (DL), which learns from extensive amounts of data. Thus, AI refers to any program that possesses the capability to solve problems, learn from experiences, and execute tasks in a similar manner to humans [86]. ASRM [87] and ESHRE [88] have already highlighted different applications of ML, such as predicting blastocyst formation from oocytes, evaluating the quality of human blastocysts, envisaging live births after embryo transfer, enhancing embryo selection, and determining optimal IVF stimulation protocols. Data-driven solutions have the potential to identify early indicators of infertility, enabling timely identification of patients who may benefit from treatment. Moreover, AI may contribute to the determination of the most suitable stimulation protocol for each patient to maximize the chances of success prior to starting treatment. This would allow the enhancement of pregnancy outcomes while reducing the time required to achieve a successful live birth.

This knowledge is of interest in planning novel approaches in the FP field in women with cancer, which could favorably solve concurrent criticisms relative to the empiricism of mature oocytes’ retrieval, cryo-preservation, IVF, and embryos’ cryostorage.

## 5. Conclusions

Loss of fertility is a major concern for female cancer survivors of reproductive-age since a common side-effect of conventional cytotoxic anti-cancer therapies is permanent damage to the ovary. Since immunotherapies are increasingly becoming a standard of care for many tumors and ICIs’ impact on gonadal function is still unknown, the employment of these drugs in the curative setting exposes more men and women of childbearing age to their adverse effects, including systemic reproductive toxicity, whose mechanism is still unknown. For this reason, considering future fertility in patients undergoing ICIs should be an important part of the therapeutic plan in young patients desiring children. As doctors, we have a responsibility to offer patients the opportunity to determine their own reproductive future. The recent discovery of OSCs within women’s ovarian cortex could provide novel options to treat the iatrogenic ovarian exhaustion resulting from anti-cancer treatments. By defining their biologic and regenerative potential, we believe that OSCs are a profitable resource of oogonial cells capable of restoring a functional oogenesis, particularly after completing cancer treatment and recovering from cancer, and also in young patients with ICI-related CTRI. In the future, we will need further research, both in multi-institutional and international settings and involving different areas of expertise to improve our understanding and management of reproductive problems related to ICIs and conceivable solutions.

## Figures and Tables

**Table 1:** Fertility preservation FP techniques available for immunotherapy-treated patients. The Oocyte/embryo cryopreservation is a well-established procedure that ensures the egg integrity, reaching around 30/40% efficiency, thus requiring hormonal stimulation with several related clinical effects on oncological patients. GnRha preserve gonadal function downregulating gonadotrophins’ production. It seems to be effective especially in breast cancer. The ovarian cortex cryopreservation allows ovaries’ endocrine function recovery and is the eligible practice for prepubertal individuals, despite the fact it is still experimental with a potential risk of cancer cell reimplantation after autologous graft. The ovarian stem cells (OSCs) from the ovarian cortex possess the ability to differentiate into mature oocytes in vitro (OLCs) under appropriate conditions. Despite this method being experimental and expert-operator dependent, it could solve ethical problems related to abnormal embryos.

Method	Purpose	Results	Pros	Cons
Embryo cryostorage	immediate oocytes fertilization	first choice among ART	optimized efficacy	ethical problems
Oocyte cryopreservation	egg integrity	best choice (with embryo cryopres.)	30–45% efficacy	2–3 week hormone stimulation
GnRHa ovarian suppression	gonadotrophins inhibition	endocrine protection	outcomes in BC: >65%	adverse iatrogenic complication
Ovarian cortex cryopreservation	avoiding COH	ovarian function recovery in >90% of cases	suitable for prepubertal females	risk of cancer cell reimplantation
Ovarian stem cells	in vitro oocytes differentiation	still experimental	genetic analysis of single cell	technician expertise

## Data Availability

Not applicable.
